# Pathological changes of renal biopsy in Sjögren Syndrome

**DOI:** 10.11604/pamj.2018.31.102.17168

**Published:** 2018-10-10

**Authors:** Nery Sablón-González, Noel Lorenzo Villalba, Yanet Parodis López, Juan Manuel Fernández, Silvia Marrero-Robayna, Melek Kechida, Rafael Camacho-Galan, Jose Carlos Rodríguez- Pérez

**Affiliations:** 1 Servicio de Nefrología, Hospital Universitario de Gran Canaria Dr Negrin, ULPGC, Spain; 2 Service de Médecine Interne, Centre Hospitalier Chrétien, Belgique; 3 Centro de Hemodiálisis Avericum, Gran Canaria, Spain; 4 Service de Médecine Interne, Hôpital Universitaire Fattima Bourguiba Monastir, Tunisie; 5 Pathology Service, Hospital Universitario de Gran Canaria Dr Negrin, Spain

**Keywords:** Sjögren syndrome, tubulointerstitial nehritis, cryoglobulins, membranoproliferative type I glomerulonephritis secondary to cryoglobulins

## Abstract

We are presenting the case of a 53-year-old woman with a history of Sjögren syndrome and a secondary antiphospholipid syndrome admitted at the Nephrology department for the evaluation of renal failure. The patient was initially diagnosed with tubulointerstitial nephritis and subsequently a membranoproliferative type I glomerulonephritis, secondary to cryoglobulins during the course of the disease. Repeated renal biopsies were required to confirm the diagnosis.

## Introduction

Sjögren syndrome (SS) is a systemic autoimmune disease with multi-organ involvement, including the kidney. The prevalence of renal involvement varies among different series, but overt renal disease is considered to be a rare manifestation. Renal involvement might be present at diagnosis or develop soon after. The most frequent SS-associated nephropathies are tubulointerstitial nephritis (TIN) and less frequently membranoproliferative glomerulonephritis (MPGN) related to cryoglobulinemia [1]. Renal biopsy is the cornerstone in the diagnosis of renal involvement in these patients. We present a 53-year-old woman with primary SS and renal failure initially in the context of TIN. With time the diagnosis was changed to a membranoproliferative type I glomerulonephritis secondary to cryoglobulins.

## Patient and observation

A 53-year-old woman with a medical history of primary Sjögren syndrome and secondary antiphospholipid syndrome was admitted to the nephrology department for renal biopsy due to worsening renal function (creatinine ranging from 1.5 to 1.9mg/dL), progressive proteinuria with a peak level of 2g/24h and microhematuria (Table 1). The renal picture was associated to arthralgias, erythematous skin lesions, and neuropathic pain in lower extremities. Her family history was noncontributory. Her current medications included prednisone 10mg daily, azathioprine 100mg twice a day, valsartan 20mg daily, carvedilol 25mg twice a day, doxazosin 8mg daily, furosemide 20mg daily, gabapentin 30mg twice a day, and amitriptyline 50mg at night. Her medical history revealed 11 years of essential hypertension and primary Sjögren syndrome diagnosed in February 2013 based on the presence of the following criteria: dry eyes and mouth > 3 months, positive Schirmer's test (without anesthesia) ≤ 5mm/5 minutes, unstimulated whole salivary flow (≤ 1.5mL in 15 minutes), and the presence of Anti-SSA (Ro) or Anti-SSB (La) antibodies. Four months later, the patient presented with paresthesias in the lower extremities and polyarthralgia. The electromyogram showed a symmetric and subacute sensitive-motor polyneuropathy. This clinical picture preceded the presence of erythematous skin lesions consistent with leukocytoclastic vasculitis. She received low doses of prednisone as well as hydroxychloroquine and gabapentin.

**Table 1:** Blood and urine laboratory measurements results as well as the different treatments during her follow-up

Year	Treatment	Renal function before treatment	Renal function after treatment	Urine sediment	Proteinuria (g/24h)
Sjogren Syndrome Antiphospholipid Syndrome 2013	Prednisone Azathiopirine Oral anticoagulation	Cr 3.67 mg/dL CKD-EPI 13.67 ml/min	Cr 1.45 mg/dL CKD-EPI 42 ml/min	Leucocytes 100 Red blood cells 50 Proteins 25	0.230 g/24 h
Sjogren Syndrome relapse 2015	Cyclophosphamide Rituximab x 2(375 mg/m2)	Cr 4.1 mg/dL CKD-EPI 17.29 ml/min	Cr 1.24 mg/dl CKD-EPI 45 ml/min	Leucocytes 100 Red blood cells 250 Proteins 500	2 g/24 h
Sjogren Syndrome and Type 2 cryoglobulinemia 2016	Metilpredinsolone 500 mg x 3 Plasmapheresis 7 sessions Cyclophosphamide 1200 mg Rituximabx2(250 mg/m2)	Cr 2.9 mg/dL CKD-EPI 17 ml/min	Cr 1.6 mg/dL CKD-EPI 35.20 ml/min	Leucocytes 100 Red blood cells 150 Proteins 500	1.4 g/24h

**Figure 1:**
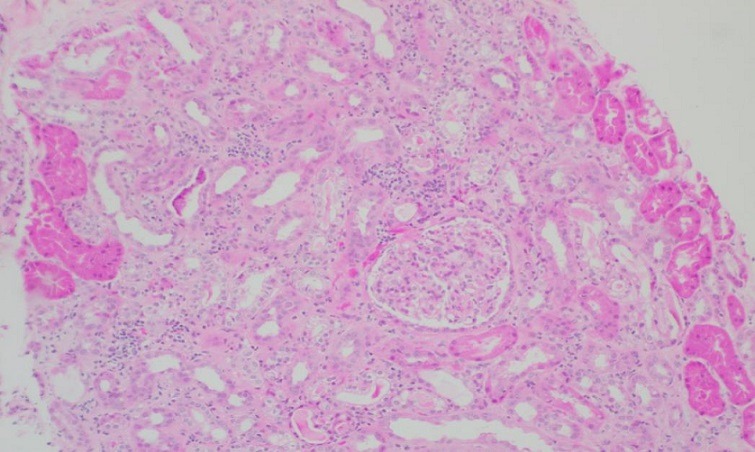
Renal parenchyma mainly shows tubulointerstitial nephritis injury (HEx100)

The patient was admitted at the nephrology department for evaluation of acute renal failure in September 2013 (creatinine reached 3.64mg/dL), normal sediment and light albuminuria. The renal ultrasound was normal. Renal biopsy report suggested a tubulointerstitial injury associated with thrombotic microangiopathy (Figure 1). The report of the histological picture shows a renal parenchyma represented by 50 glomeruli, one in global sclerosis. Although some glomeruli appear congestive, only capillary wall thickening changes are seen in a minority of them and in one fibrin thrombus. A marked intimal fibrosis is seen in the arteries of greater caliber, with significant reduction of the light, one of them with signs of recanalization. Fibrinoid necrosis is not observed. The tubulointerstitial component shows tubulointerstitial nephritis with scarce inflammatory infiltrate that is mostly chronic, mild tubulitis and marked epithelial reactive nuclear changes with hydropic changes of the cytoplasm, many of them thinned. Some protein cylinder is appreciated. The study with immunofluorescence (11 glomeruli) was negative except traces of complement in some glomeruli. She was treated with azathioprine and prednisone. At discharge, renal function was compatible with chronic kidney disease stage 3a. The patient remained clinically stable until the end of 2014 when she presented with worsening of the neurological manifestations, ulcerated skin lesions, and hematochezia. Laboratory tests revealed microhematuria and worsening of renal function (creatinine ranging from 1.6 to 2.9mg/dL). She was re evaluated by the rheumatologist who prescribed one isolated bolus of cyclophosphamide and started weekly rituximab. After two doses of rituximab, the patient presented with a clinical picture consistent with serum sickness. During this acute episode, worsening of renal failure (creatinine reached 4.1mg/dL) was noted. The previous treatment was discontinued; she was put on azathioprine and prednisone 1mg/kg/day. Creatinine went down to 1.5mg/dL but microhematuria and albuminuria (maximum 200mg/day) persisted. Azathioprine was discontinued and prednisone dose was progressively reduced. The findings of a second renal biopsy were similar to that of 2013.

At this admission, the physical examination revealed ankle edema and irregular ulcerated crusted lesions in the lower extremities. These lesions were infiltrative and painful. The cardiorespiratory system examination was normal as was the abdominal exam. Laboratory studies showed the following values: blood urea nitrogen 93mg/dL; serum creatinine 2.97mg/dL (corresponding to estimated glomerular filtration rate [eGFR] of 17.29mL/min/1.73m2 as calculated by CKD-EPI); uric acid 9.54mg/dL; serum osmolarity 302mOsm/L; hemoglobin 9.1g/dL, corpuscular volume 85fL; serum albumin 5.7g/dL and type II cryoglobulins 4.8%. Urinalysis showed proteinuria (500mg/dL), 250 red cells per uL, piuria, and bacteriuria. Urinary protein was 1.4 g/24h. Urine sodium was 48mEq/L; and urinary potassium was 8.7mEq/L. The EFNa was 0.8%. Serum electrolytes and liver and thyroid function tests were normal. An autoimmune panel revealed positivity for antinuclear antibodies, rheumatoid factor, and anti-SS-B and A antibodies. Complement C3 was 30mg/dL and C4 was 4mg/dL (normal values 88 to 201mg/dL and 15 a 45mg/dL respectively). Serum electrolytes and liver and thyroid function tests were normal. Serology for HIV, HBV, and HCV came back negative. Serum and urine protein electrophoresis showed no protein spike. A total body tomography ruled out the presence of adenopathy or visceromegaly.

**Figure 2:**
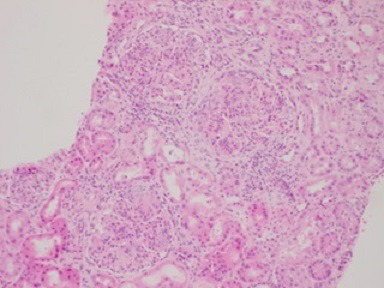
Lobular pattern of the glomeruli (HEx100)

**Figure 3:**
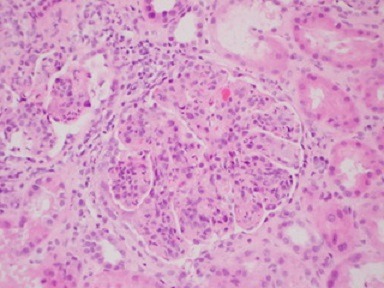
Lobular pattern of the glomeruli (HEx200)

**Figure 4:**
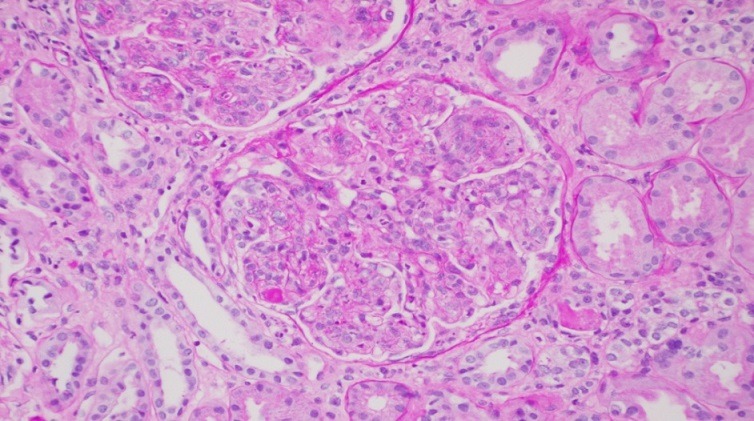
PAS staining (PASx200)

**Figure 5:**
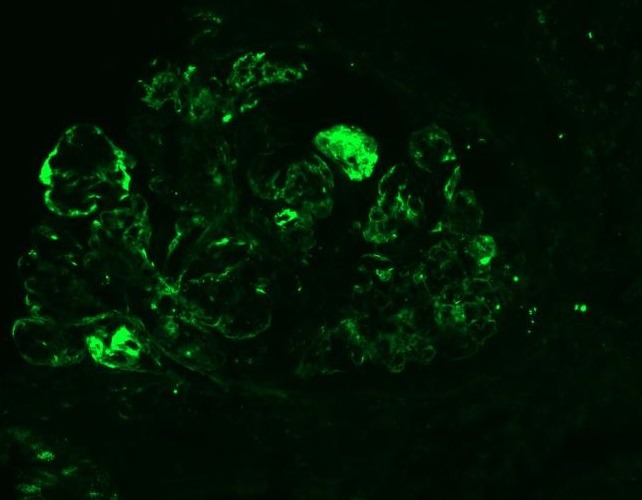
Immunofluorescence C3 studies (x200)

**Figure 6:**
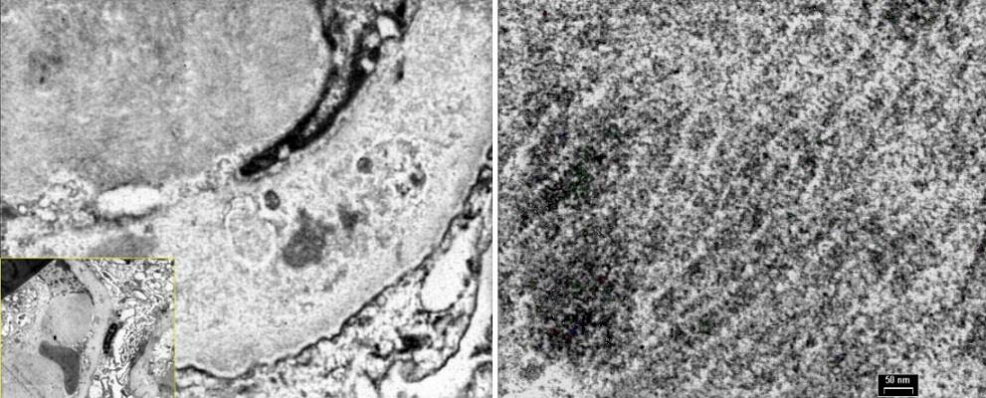
A) capillary membranes were irregularly thickened and electron-dense intramembranous deposits (x5600); B) the tubular structures of 26 nanometers (x36000)

A third renal biopsy showed 51 glomeruli, 4 with global sclerosis. The glomeruli presented a diffuse lesion (Figure 2) with a lobular pattern due to intense mesangial cell proliferation and obliteration of the capillary lumen by mesangial, polymorphonuclear, and macrophage cells (Figure 3). Three glomeruli presented with capillary thrombi and extracapillary proliferation. The interstitium showed 15% interstitial fibrosis and tubular atrophy and abundant chronic lympho-plasmo-histiocytic inflammation consistent with tubulointerstitial nephritis. PAS staining (Figure 4) showed diffuse and global glomerular lesions with a type 1 membranoproliferative pattern, showing mesangial increase of the matrix and its cells, with contracting the capillary lumens as well as the presence of macrophages, all of which causes a marked accentuation of the lobular pattern of the glomerular ball. The presence of capillary thrombi and extracapillary proliferation is added. Immunofluorescence showed intense diffuse positivity for capillary and moderate C3 (Figure 5), focal IgM, and IgG with traces of kappa and lambda. The ultrastructural evaluation showed 4 glomeruli with capillary lights occupied by cytoplasm of endothelial cells and macrophages. Numerous capillary membranes were irregularly thickened. Electron-dense subendothelial deposits were noted. At high magnification, grouped tubular structures of 26 nanometers with intratubular light were frequently found in the capillary light. Amorphous electron-dense deposits were observed in the subendothelial, intramembranous, and subepithelial position as well as fusion of several pedicels. Mild to moderate tubular atrophy and interstitial tubular fibrosis were observed (Figure 6A, Figure 6B). All of these findings were consistent with a Cryoglobulinemic glomerulonephritis with a pattern of membranoproliferative type I.

## Discussion

Sjögren syndrome is a chronic, slowly-progressive autoimmune disorder characterized by lymphoplasmacytic infiltration of the exocrine glands, mainly the salivary and the lacrimal glands. The syndrome could present as an isolated condition or in association with other autoimmune conditions as in the case presented [1]. The clinical spectrum is wide; patients generally present with symptoms related to xerostomia and keratoconjunctivitis but they may also present with pulmonary, neurological, cardiovascular, and kidney manifestations. The prevalence of renal involvement in Sjögren syndrome ranges from 2 to 67 percent [1-3]. This wide variation probably results from the different classification criteria used in the studies, as well as the selection of the patients. In this respect, TIN is the most common kidney manifestation in Sjögren syndrome and results from interstitial lymphoplasmacytic inflammation [4, 5].

TIN is most often manifested by tubular acidosis but also by hypokalemia and/or hypophosphatemia and might evolve to renal insufficiency [6]. Tubular acidosis is the consequence of the inability of the distal renal tubule to secrete hydrogen ions. Hyposthenuria has also been described and it is due to an abnormality in the urine concentration mechanism [6, 7]. Chronic renal impairment is associated with tubular atrophy and interstitial fibrosis. The presence of tubular granulomata has also been reported mostly in patients presenting with other conditions such as sarcoidosis. Glomerular involvement is less often seen [7]. This may include membranoproliferative/cryoglobulinemic glomerulonephritis (GN), membranous nephropathy, mesangioproliferative GN, and focal segmental glomerulosclerosis [8, 9]. In terms of renal prognosis, those patients presenting with TIN have a more favorable prognosis compared with those with GN. Poor prognosis in patients with GN is probably related to mixed cryoglobulinemia and the risk of lymphoma in cryoglobulinemic GN [8, 9]. Our patient presented in 2013 with a clinical picture consistent with leucocytoclastic vasculitis in the course of a Sjogren syndrome with cryoglobulinemia, but cryoglobulins were negative, and the renal biopsy was compatible with tubulointerstitial nephritis.

These results would have been carefully evaluated as laboratory testing of cryoglobulins is fraught with false-negative results due to suboptimal specimen collection and handling. Therefore, repeating testing must be done when this condition is suspected. This may explain why cryoglobulin testing came back negative even though the clinical picture strongly suggested their presence. Despite this, we wish to emphasize that only a subset of patients with circulating cryoglobulins develope symptoms related to tissue damage. This proportion of patients varies depending on age, underlying illness, and type of cryoglobulins. The decision of performing a third renal biopsy was largely discussed considering the previous results. As in the case presented, renal lesions are observed in association with systemic manifestations although they may occasionally be isolated. Sjögren syndrome is the most common cause of non-HCV-related mixed cryoglobulinemia, followed by other autoimmune diseases (systemic lupus erythematosus and rheumatoid arthritis). The current patient was double positive for cryoglobulin and rheumatoid factor; their activities were both found to be crucial for skin involvement, whereas only the cryoglobulin activity was necessary for renal injury [10]. The evolution of renal function and the clinical context despite the previous renal biopsy led us to repeat the renal biopsy which confirmed the diagnosis.

## Conclusion

Renal involvement is rare in Sjögren Syndrome but its diagnosis is important for the renal outcome. Regarding the disease course, renal biopsy might be repeated if necessary and not assume the initial diagnosis. Thus, proper diagnosis and adequate treatment could be assessed.

## Competing interests

The authors declare no competing interest.
